# Ancient origin of the divergent forms of leucyl-tRNA synthetases in the Halobacteriales

**DOI:** 10.1186/1471-2148-12-85

**Published:** 2012-06-13

**Authors:** Cheryl P Andam, Timothy J Harlow, R Thane Papke, J Peter Gogarten

**Affiliations:** 1 Department of Molecular and Cell Biology, University of Connecticut, 91 North Eagleville Rd, Storrs, CT, 06269-3125, USA

## Abstract

**Background:**

Horizontal gene transfer (HGT) has greatly impacted the genealogical history of many lineages, particularly for prokaryotes, with genes frequently moving in and out of a line of descent. Many genes that were acquired by a lineage in the past likely originated from ancestral relatives that have since gone extinct. During the course of evolution, HGT has played an essential role in the origin and dissemination of genetic and metabolic novelty.

**Results:**

Three divergent forms of leucyl-tRNA synthetase (LeuRS) exist in the archaeal order Halobacteriales, commonly known as haloarchaea. Few haloarchaeal genomes have the typical archaeal form of this enzyme and phylogenetic analysis indicates it clusters within the Euryarchaeota as expected. The majority of sequenced halobacterial genomes possess a bacterial form of LeuRS. Phylogenetic reconstruction puts this larger group of haloarchaea at the base of the bacterial domain. The most parsimonious explanation is that an ancient transfer of LeuRS took place from an organism related to the ancestor of the bacterial domain to the haloarchaea. The bacterial form of LeuRS further underwent gene duplications and/or gene transfers within the haloarchaea, with some genomes possessing two distinct types of bacterial LeuRS. The cognate tRNA^Leu^ also reveals two distinct clusters for the haloarchaea; however, these tRNA^Leu^ clusters do not coincide with the groupings found in the LeuRS tree, revealing that LeuRS evolved independently of its cognate tRNA.

**Conclusions:**

The study of leucyl-tRNA synthetase in haloarchaea illustrates the importance of gene transfer originating in lineages that went extinct since the transfer occurred. The haloarchaeal LeuRS and tRNA^Leu^ did not co-evolve.

## Background

The archaeal order Halobacteriales, commonly known as haloarchaea, consists of extremely halophilic, aerobic or facultative anaerobic organisms currently classified into 29 genera (http://www.the-icsp.org/taxa/halobacterlist.htm) These organisms are the dominant taxa in hypersaline ecosystems, such as salterns, salt and soda lakes and coastal areas, in which NaCl concentrations can reach 150–350 g/L [1]. Members of the Halobacteriales are known to undergo frequent HGT and recombination [2-4]. The recently identified methylaspartate cycle for acetyl-CoA assimilation in haloarchaea consists of enzymes acquired through HGT. The pre-existing genes acquired from different bacterial donors were originally involved in various metabolic processes [5]. Analyses of the bacteriorhodopsin and halorhodopsin proteins in the haloarchaea also suggest that HGT has played a role in their evolution [6].

The Halobacteriales are usually considered to have evolved from a group of halophilic methanogens. Phylogenies based on rRNA, concatenated proteins, and proteins involved in transcription and translation show the Halobacteriales as a sister group to the Methanosarcinales [7,8]. However, whole-genome-based phylogenetic reconstructions often placed them at the base of the archaeal domain [9,10], which might reflect the high number of genes in the haloarchaea that are of bacterial origin. Alternatively, they could be from extinct archaeal lineages that left a “fossil” in the molecular record.

Very few studies have provided evidence for ancient transfers from now-extinct lineages that existed prior to or during the time of last universal common ancestor (LUCA) of all life, or of each three domains. An example is the case of the rare pyrrolysyl-tRNA synthetase (PylRS) that charges the tRNA^Pyl^ with the non-canonical amino acid pyrrolysine (Pyl) [11]. This rare enzyme has a restricted distribution, to date found only in members of the archaeal order Methanosarcinales, the firmicute *Desulfitobacterium hafniense* and a Deltaproteobacterium endosymbiont [12]. In relation to the other aaRS, PylRS is placed as a deep-branching lineage within the aaRS subclass IIb, emerging prior to the most recent common ancestor of the bacterial and archaeal/eukaryal domains [13]. The phylogenetic distribution of this enzyme suggests that these extant taxa acquired PylRS through several HGT episodes from an ancient, most likely extinct, lineage [12]. A rare form of seryl-tRNA synthetase (SerRS), to date only found in some methanogens, based on phylogenetic reconstruction was suggested to have been acquired through HGT from a deep branching lineage [14]. The patchy distribution of another uncommon Class II aaRS, O-phosphoseryl-tRNA synthetase (SepRS), is also indicative of ancient HGT. SepRS charges tRNA^Cys^ with O-phosphoserine (Sep), a precursor of cysteine (Cys), to form Sep-tRNA^Cys^ and is then converted to Cys-tRNA^Cys^[15]. Phylogenetic analyses show that SepRS predates the duplication event that gave rise to the two phenylalanyl-tRNA synthetases (PheRS) subunits and also arose before the divergence of the organismal LUCA [16].

A challenge in the analyses of genetic contributions of ancient lineages to existing genomes is the absence of information about donor lineages because majority of them are already extinct. Genes that arose prior to the time of LUCA are expected to exhibit high divergence from their homologs. This would reflect an extremely long coalescence time to a most recent common molecular ancestor, occurring well before the organismal common ancestor [17]. In the case of the PylRS [12], the rare form of SerRS [14], and SepRS [16], we can infer from their phylogenetic histories that they likely were already present prior to or during the time of the organismal LUCA.

In this study, we show the existence of two forms of LeuRS in the Halobacteriales that arose through ancient HGT. The bacterial form of LeuRS in the haloarchaea was likely acquired from a relative of the ancestor of the bacterial domain and further underwent gene duplication, transfer and divergence within the haloarchaea. We also discuss the impact of ancient HGT events in generating genetic diversity in present-day lineages.

## Results and discussion

### Two major clades of Halobacteriales in the LeuRS phylogeny

aaRS are ancient enzymes that catalyze the attachment of tRNA with its cognate amino acid during the translation process. This function is essential in maintaining the fidelity of the genetic code and all 20 aminoacyl-tRNA species are essential for all living organisms. Although aaRSs are part of the conserved "information processing and storage" gene set, aaRS are frequently transferred across species boundaries and even between domains [18-20], most likely due to the limited interactions with other biomolecules [18].

**Figure 1:**
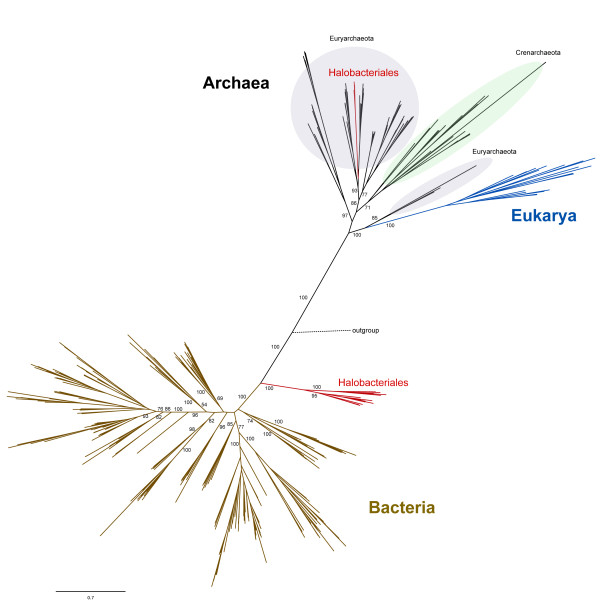
**Maximum likelihood phylogenetic tree of LeuRS across the three domains of life.** Numbers on the nodes indicate bootstrap support. Only the support values for major clades are shown for clarity. Members of the Halobacteriales are highlighted in red. The tree was rooted using amino acid sequences of isoleucyl-tRNA synthetase from *Thermotoga maritima, Aquifex aeolicus, Aeropyrum pernix* and *Methanopyrus kandleri*. Details of this tree are shown in Additional file 1: Figure S1.

Phylogenetic reconstruction using the amino acid sequences of LeuRS from Bacteria, Archaea and Eukarya shows the expected canonical pattern of having the archaeal and bacterial versions as distinct clusters, and the archaeal and eukaryal clades as sister groups (Figure 1). Within the Archaea, the two major phyla, Crenarchaeota and Euryarchaeota, can be distinguished (the other proposed archaeal phyla are not labeled; see Additional file 1: Figure S1 for their phylogenetic position). The LeuRS tree shows clustering of sequences into major phyla that suggests an evolutionary history largely dominated by vertical inheritance (Additional file 1: Figure S1.

**Figure 2:**
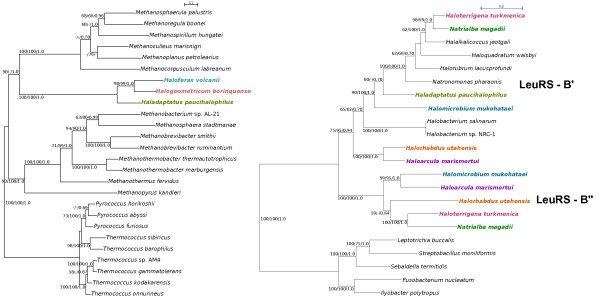
**Phylogenetic analyses of the two LeuRS forms found in Halobacteriales.** A detailed look at the haloarchaeal forms of LeuRS–A (left panel) in colored text and the bacterial type (LeuRS–B, right panel) in gray boxes. Haloarchaeal sequences represented in the same color indicate genomes that carry two types of LeuRS. Numbers on the branches indicate bootstrap support under maximum likelihood (left) and distance analyses (middle), and posterior probabilities (right). Only bootstrap values above 50% and posterior probabilities above 0.50 are shown.

The existence of two distinct groups of Halobacteriales in this LeuRS phylogeny is noteworthy. A smaller group of haloarchaea clusters within the Euryarchaeaota as expected [8,21,22] and a larger group is located at the base of the bacterial domain (Figure 1). We refer to the archaeal version of LeuRS in Halobacteriales as LeuRS-A and the bacterial version as LeuRS-B (*cf.* Figure 2). The extremely deep branch of the larger Halobacteriales clade relative to the rest of the Bacteria suggests an ancient horizontal acquisition of *leuS* from an unknown source, most likely from a relative of the ancestor of the Bacteria, to the Halobacteriales. The donor and the recipient may not have lived at the same time, and the transfer might have involved an intermediate carrier.

A single protein can contain parts that differ in phylogeny and substitution rates. We used GARD (Genetic Algorithm for Recombination Detection [23]) to investigate if different parts of the LeuRSs in haloarchaea have different histories. Using MUSCLE [24] and SATé [25] alignments, GARD determined breakpoints corresponding to position 780 (MUSCLE) and 628 (SATé) in the *Halogeometricum* LeuRS sequence, respectively. Further inspection of the multiple sequence alignment revealed that most of the phylogenetic information distinguishing the archaeal and bacterial type LeuRSs is contained in the larger amino terminal part of the alignment. This part contains the domain that catalyzes the esterification between leucine and tRNA, and contains many positions universally conserved between the domains. The carboxy terminal part of the alignment encodes the tRNA recognition domain. While GARD found a significant difference between the tree topologies determined for the two parts of the multiple sequence alignment, in both phylogenies reconstructed separately for the two parts the SATé alignment, the LeuRS-B sequences group at the base of the bacterial homologs, whereas LeuRS-A group with the euryarchaeal homologs (see Additional file 2: Figure S2). The role of the two parts of LeuRS in interacting with tRNA^Leu^ are illustrated in Additional file 3: Figure S3. Using the breakpoint from the GARD analysis of the MUSCLE alignment resulted in a carboxy terminal portion that was too short for reliable phylogenetic reconstruction. It is noteworthy that in the maximum likelihood phylogeny for this short fragment all haloarchaea grouped together, albeit with a bootstrap support value of only 47%. As most of the haloarchaeal fragments failed a chi-square test for compositional homogeneity, this finding may reflect a shared compositional bias in the haloarchaeal sequences, although the possibility that the carboxyterminal part of LeuRS might have a different evolutionary history from the rest of the enzyme cannot be excluded.

To explore the possibility that placement of the haloarchaeal LeuRS-B reflects an artifact created through long branch attraction, we calculated the pairwise distances between representatives of the bacterial LeuRS (*Salinibacter ruber* and *Halanaerobium prevalens*), archaeal LeuRS (*Haloferax volcanii, Halogeometricum borinquense, Methanocorpusculum labreanum, Pyrococcus furiosus*), haloarchaeal LeuRS-B (the two LeuRS-B copies in *Halomicrobium mukohataei* and *Haloterrigena turkmenica*) and the outgroup (Isoleucyl-tRNA synthetase from *Methanopyrus kandleri* and *Thermotoga maritima*). Mean pairwise distances from the outgroup do not show significant differences (0.5364 ± 0.0511 for the archaeal LeuRS, 0.3915 ± 0.0268 for the bacterial LeuRS, and 0.4038 ± 0.0791 for the haloarchaeal LeuRS-B). Analysis of compositional homogeneity using chi-square test as implemented in the program TREE-PUZZLE [24] indicated that the LeuRS-B sequences do not have atypical composition (P > 0.05). We do not find evidence that the placement of haloarchaeal LeuRS-B at the base of the bacterial homologs is due to an artifact created by these sequences being more divergent or having a different composition, and we find no indication of a close association of Halobacteriales LeuRS-B sequences with any specific bacterial or archaeal group. Nevertheless, artifacts created in the alignment certainly have the potential to increase apparent support values, thus a placement of the LeuRS-B sequences within the cluster of bacterial homologs cannot be excluded.

We performed more detailed phylogenetic analyses of the two haloarchaeal clusters and their closest relatives to determine the phylogenetic relationships among the members of each group (Figure 2). We analyzed 14 haloarchaeal genomes that were available in the NCBI completed microbial genome database. Out of these, only three genomes carry the LeuRS-A form – *Haloferax volcanii, Halogeometricum borinquense* and *Haladaptatus paucihalophilus*. Their sequences show close affinities to members of the Methanomicrobiales and Methanobacteriales (Figure 2a). The bacterial version LeuRS-B exhibits a more complicated picture (Figure 2b). Two highly-supported clusters can be observed, which we refer to as B’ and B”. In five of the genomes included in this study (*Natrialba magadii, Haloterrigena turkmenica, Halomicrobium mukohataei, Haloarcula marismortui* and *Halorhabdus utahensis*), both B’ and B” are present*.* Two possible scenarios can explain the observed distribution of LeuRS-B. The observation that B' and B'' group together at the base of the bacteria indicates their divergence occurred either in the donating lineage, or following the transfer. The two distinct scenarios are (a) the B form was already present in the haloarchaeal ancestor; versus (b) the B form was later acquired, but spread to different haloarchaeal groups through biased gene transfer [14].

**Figure 3:**
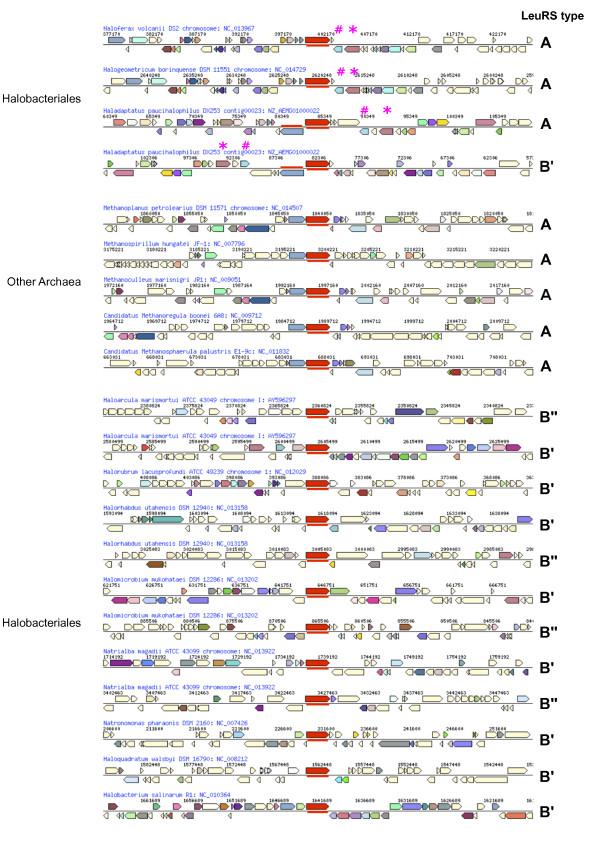
**Genomic neighborhood of*leuS*in the Halobacteriales and other Archaea.** The central red arrow represents the gene encoding LeuRS. The type of LeuRS is indicated on the right hand side of each gene neighborhood. Genes depicted in the same color (except light yellow and white) are from the same cluster of orthologous genes (COG), light yellow and white arrows indicate genes without COG assignment and pseudogenes, respectively. While the gene neighborhood of the archaeal type LeuRS is conserved in the depicted haloarchaea and in methanogenic archaea (but not between the two groups), the gene neighborhood of type B LeuRS appears less conserved, revealing frequent non-homologous recombination and rearrangements. See text for further discussion. Genes flanking *leuS* marked with # and * refer to alanine dehydrogenase and a thermosome subunit, respectively.

Supporting evidence for the second scenario is observed in the genomic region around B’ and B”. The two B forms do not sit in the same genomic neighborhood and do not exhibit synteny in Halobacteriales species that possess the B form (Figure 3). Also, genes flanking the B’ form are not conserved among the different organisms carrying the B’ and the same is true for the gene neighborhood of B”. In contrast, genomic neighborhoods of LeuRS-A demonstrate synteny in terms of gene identity and order. Methanogenic archaea also reveal synteny for their gene coding for LeuRS, suggesting that the A form has undergone vertical transmission and/or gene transfer followed by homologous recombination. The B form of the enzyme, however, appears to have been transferred among the Halobacteriales species involving non-homologous recombination into different parts of the recipients’ genomes. If a second LeuRS is integrated into a genome by non-homologous recombination, following a period of coexistence, one of the two homologs may eventually be lost. If the distribution of the two LeuRS-B forms had been generated through gene loss alone, we would expect to see syntenic regions around the gene coding for the B’ form and syntenic regions around the gene coding B”, and these two regions would be distinct from each other. While we do not detect any synteny in our sample of LeuRS-B forms, we cannot rule out the alternative explanation that genomic regions encoding the LeuRS-B forms experienced more frequent rearrangements than regions harboring the LeuRS-A forms.

A second line of support for HGT of the two B forms comes from parametric bootstrapping analysis as implemented in LGT3State [26]. In this test, the null model requires that HGT is absent in the evolution of the LeuRS-B genes and that gene loss events can explain the distribution patterns. This model implies that the most recent Halobacteriales ancestor carried both types of LeuRS-B. The second model assumes gene losses and gains of the alternative forms can occur, that is, a genome carrying LeuRS-B’ can gain the LeuRS-B”, and vice versa, resulting in a genome with both types present, from which one type may eventually be lost. Using the LGT3State program [26], we generated 1000 bootstrap distributions under the gene loss only model. Thus, we have 1000 datasets reflecting the outcomes under the null model, which are compared to the real data. The distribution of the 1000 likelihood values gives us a measure of what to expect under the null hypothesis. The log-likelihood values obtained for the bootstrapped samples evaluated under the HGT model ranged from −43.2 to −49.6, and are much lower than the log-likelihood values when assuming the HGT model for the original tree (−6.35). Hence, we can reject the gene loss only model with a significance level of P<0.001.

Interestingly, we also observed that no genome possesses only the B” form (Figure 2b), i.e., B” is always found to co-exist with the B’. For the genomes that carry the two B copies, maintenance of the two functionally identical enzymes likely confers a selective advantage to the host. In bacteria, differential sensitivity of multiple copies of aaRS with redundant functions may benefit the organism against naturally occurring antibiotics [27]. The antibiotic capabilities of Archaea have only recently been investigated. Peptide antibiotics produced by some members of the Archaea, referred to as archaeocins, have been identified from haloarchaea and *Sulfolobus* and were reported to exhibit cross-kingdom toxicity [28]. A recent study showed that methanogenic archaea exhibit differences in susceptibility to various antibiotics, such as ampicillin, streptomycin, gentamicin, rifampicin, ofloxacin, tetracycline [29]. It is also possible that there is a difference in the functional efficiency of the two LeuRS-B forms, with B” being less efficient in aminoacylating some of its cognate tRNAs. This may be similar to the intragenomic heterogeneity in the ribosomal operons of *Haloarcula marismortui*, which exhibit differences in gene expression under different environmental conditions [30]. Alternatively, the functioning enzyme may consist of a B'B'' heterodimer, allowing more degrees of freedom to accommodate destabilizing mutations [31], as observed in *Aquifex aeolicus*[32,33]; the transition from a homo- to a heterodimer initially might not have been adaptive, but the resulting heterodimer nevertheless may be under strong purifying selection [34]. However, the latter scenario is unlikely as the genes encoding the B' and B" forms are located in different parts of the genomes (Figure 3).

*Haladaptatus paucihalophilus* possesses both the A and the B’ form of LeuRS (Figure 2). Both copies are located adjacent to each other and are divergently transcribed. Two of its flanking genes (coding for a thermosome subunit and alanine dehydrogenase) are also found in the genomic neighborhood of *leuS* in the other two haloarchaea that possess only the A form (*Haloferax volcanii* and *Halogeometricum borinquense*; Figure 3). This is compatible with the scenario that *Haladaptatus* originally had the A form and has subsequently acquired the B’ form through HGT from another haloarchaeon.

The archaeal and bacterial forms of LeuRS are significantly distinct from each other (Additional file 4: Table S1). The identities between the A and B forms range from 21-26%, reflecting the very deep divergence that gave rise to these two forms. In contrast, the two LeuRS-B forms exhibit 46–53% identity between the two B-types suggesting a more recent divergence event.

### Scattered distribution of the different LeuRS in the Halobacteriales

Previous studies have reported the challenge of using the 16S rRNA phylogeny to determine the evolutionary relationships of the Halobacteriales [35]. Two factors have been implicated: the presence of multiple divergent copies of this gene in a single genome in many haloarchaeal species and that recombination of the rRNA gene occurs frequently between species [36]. Paralogous copies of rRNA operons in these organisms have been reported to show more than 5% divergence [35], and identical sequences have been found in strains that are otherwise clearly differentiated, making it difficult to establish accurate Halobacteriales relationships.

**Figure 4:**
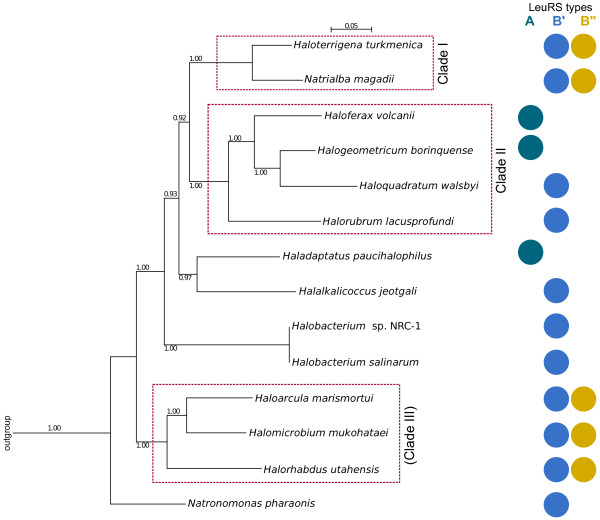
**Phylogenetic analyses of the concatenated housekeeping proteins in the Halobacteriales (referred to as MLSA tree; adapted from.**[39]**).** Numbers on the branches give posterior probabilities. The outgroup species used were *Methanosarcina acetivorans, Methanosarcina barkeri, Methanosarcina mazei, Archaeoglobus fulgidus, Methanothermobacter thermautotrophicus* and *Methanococcus vannielli*, similar to what was used in [39]. Only posterior probabilities above 0.50 are shown.

In light of the problems posed by using 16S rRNA sequences in haloarchaeal phylogeny, alternative markers have been used to establish relationships within the Halobacteriales. The RNA polymerase subunit B’ (RpoB’) has been put forward to be a more useful alternative [37,38], but it is also subject to HGT. More recently, the multilocus sequence analysis (MLSA) approach has been demonstrated to effectively discriminate among strains and species in the Halobacteriales [39]. Using this method, we concatenated the amino acid sequences of five housekeeping proteins from the 14 Halobacteriales species that we used in the LeuRS phylogeny. Phylogenetic reconstruction revealed the two highly supported clades (Figure 4), similar to the results of [39]. In the MLSA tree in our study, Clade I consists of *Haloterrigena* and *Natrialba*, while Clade II is comprised of *Halogeometricum, Haloquadratum, Haloferax* and *Halorubrum* (Figure 4). We also obtained another highly supported group, consisting of *HaloarculaHalomicrobium* and *Halorhabdus* (Figure 4). For the purposes of this study, we will refer to the third group as clade III. This phylogeny is also similar to one obtained from concatenated ribosomal proteins (Williams, Gogarten, Papke, personal communication) and the phylogeny inferred from a 3,853 gene supermatrix [40]. In particular, the three major groups of haloarchaea were also identified in these studies.

Mapping the presence and absence of the three LeuRS in the MLSA tree shows that all species belonging to clades I and III possess both B forms of the bacterial LeuRS. Given that LeuRS genes were frequently transferred within the haloarchaea, we do not interpret the co-occurrence of the B' and B" forms as shared derived character for clade I and III. For the archaeal version (LeuRS-A), we observed a dispersed distribution, mostly in branches that appear to have diverged more recently. If we consider the MLSA tree as a suitable representation of the species phylogeny of this group, and only take into account the distribution of LeuRS types within this group, then the initial assumption would be that the ancestor of the Halobacteriales possessed the bacterial form of LeuRS. However, another more likely scenario is that the presence of the archaeal version of the enzyme (LeuRS-A) is the ancestral state in the Halobacteriales. The clustering of the haloarchaeal LeuRS-A cluster within the euryarchaeal homologs, specifically with those from methanogens, would indicate shared ancestry [21,22], and the archaeal LeuRS would be vertically inherited by the Halobacteriales. The single divergence event that gave rise to the B' and B'' forms likely took place early in the evolution of the Halobacteriales, followed by the spread or retention of both forms of LeuRS-B within the order.

Assuming that the Halobacteriales ancestor originally possessed the archaeal form acquired through vertical inheritance from the common ancestor of all Archaea, it later on gained the bacterial LeuRS through horizontal transfer from a deep branching bacterial lineage, possibly still unsampled or now extinct. The finding that the haloarchaeal LeuRS-B diverged before the homologs found in bacteria suggests that either the lineage donating LeuRS-B to the haloarchaea or the haloarchaea themselves coexisted with the bacterial most recent common ancestor. More than one lineage could have carried the bacterial version of LeuRS before it was transferred to the haloarchaea; however, provided that the deep branching of the haloarchaeal LeuRS form B is not an artifact, all the scenarios imply that the bacterial version now residing in the haloarchaea, coexisted with the ancestor of the bacterial domain. Following transfer to the haloarchaea, the bacterial form spread among the majority of the Halobacteriales through vertical inheritance and HGT biased toward close relatives [14,41], with some species possessing one form while in others, both forms of the bacterial LeuRS are retained.

We then compared the LeuRS-A (Figure 2a) and LeuRS-B (Figure 2b) with the MLSA tree (Figure 4) to see if there are any conflicting topologies between them. For LeuRS-A, we observed similarity regarding the placement of the three species. *Haloferax* and *Halogeometricum* group together, and *Haladaptatus* is found at the base (Figure 2a). The topology of the LeuRS-B” tree was also similar to the MLSA tree, except for the placement of *Halorhabdus* (Figure 2b). This, however, is not highly supported and therefore we cannot draw any conclusion from it. In LeuRS-B”, the groupings of *Natrialba* and *Haloterrigena*, and of *Haloarcula* and *Halomicrobium* are similar to what we found in the MLSA tree. In comparing the LeuRS-B’ and the MLSA tree, we also observed the same clustering of the above mentioned two pairs of haloarchaea. An important conflict, however, is the phylogenetic position of *Halomicrobium*; the MLSA tree places it in clade III, while in the LeuRS-B’ tree, its position is highly supported at the base of the clade II (Figure 2a). Within clade III of the MLSA tree, *Haloarcula* and *Halomicrobium* have a closer relationship than with *Halorhabdus*. Hence, the LeuRS-B’ topology indicates a transfer from clade II to *Halomicrobium*. Another possible conflict is that of *Natronomonas*, which clusters with the clade II species in the LeuRS tree.

Topologies of the MLSA tree and each of the LeuRS trees indicate that (1) the Halobacteriales came to possess the archaeal form through common ancestry with the rest of the Archaea that was eventually lost in a majority of the Halobacteriales, and (2) the bacterial LeuRS types were vertically and horizontally inherited within the group. We can be certain that at least one HGT event took place – the transfer from a deep branching, currently unsampled bacterial lineage diverging most likely before the bacterial common ancestor to the Halobacteriales.

### Archaeal tRNA^Leu^ phylogeny shows two groups of haloarchaea

**Figure 5:**
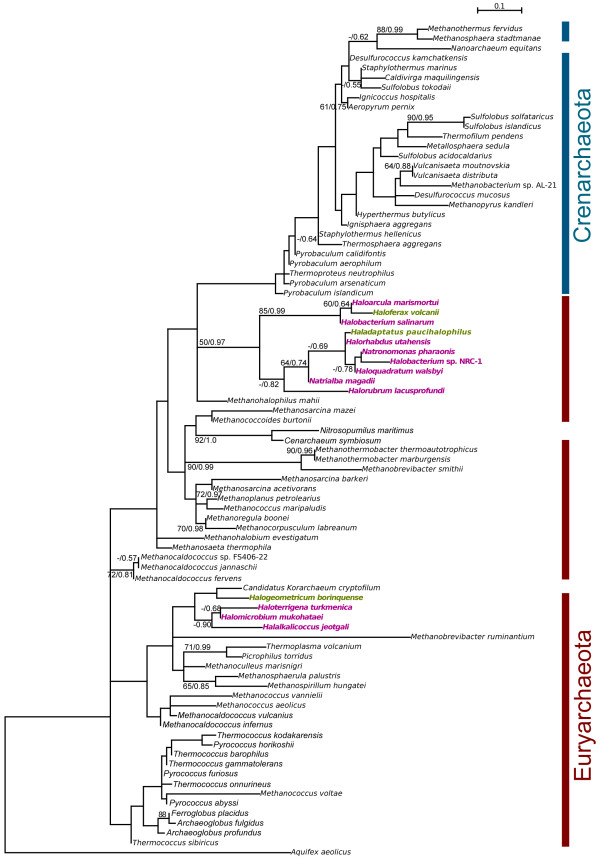
**Phylogenetic analyses of the tRNA**^**Leu**^**in Archaea.** Numbers on the nodes indicate bootstrap support under maximum likelihood (left) and posterior probabilities (right). Members of the Halobacteriales that possess the archaeal version of LeuRS are highlighted in green and those with only LeuRS-B are in pink. The tree was rooted using the sequence from *Aquifex aeolicus*. Only bootstrap values above 50% and posterior probabilities above 0.50 are shown.

Transfer RNAs (tRNAs) are considered to be one of the primordial molecules that arose in the RNA world before protein biosynthesis emerged on Earth. They are a critical component in the translation machinery, linking their anticodon triplet between the mRNA and amino acid. To determine if the divergence of LeuRS influenced the evolutionary route of their cognate tRNA, phylogenetic reconstruction of the archaeal tRNA^Leu^ sequences was performed (Figure 5). We did not obtain high bootstrap support for the tRNA^Leu^ tree due to the short sequences of tRNA molecules. The length of canonical tRNA sequences is only about 76 nucleotides [42] and this does not provide sufficient phylogenetic information for a well-resolved phylogeny. However, both maximum likelihood and Bayesian methods revealed similar results.

Superficially similar to the LeuRS tree, two main groups of Halobacteriales are found in the tRNA^Leu^ tree (Figure 5). However, the distribution of the haloarchaea into the two groups differs significantly from that found in their corresponding synthetase tree. In the LeuRS tree, the smaller group of Halobacteriales consists of *Haloferax*, *Halogeometricum* and *Haladaptatus*, and the majority is found in a bigger cluster distinct from it (Figure 2). In contrast, the three genera mentioned above do not group together in the tRNA^Leu^ tree (Figure 5). One cluster consists of *Haloferax* and *Haladaptatus*, together with *Haloarcula, Halobacterium, Halorhabdus, Natronomonas, Haloquadratum, Natrialba* and *Halorubrum*. A second cluster is comprised of *Halogeometricum, Haloterrigena, Halomicrobium* and *Halalkalicoccus*.

The discovery of the conflicting groupings of haloarchaea in the LeuRS and the tRNA^Leu^ phylogenies begs the question of the evolution regarding LeuRS-tRNA^Leu^ metabolic interaction in these organisms. Our results suggest that the evolutionary route that the haloarchaeal tRNA^Leu^ took was independent of the evolution of the aaRS that aminoacylates it. This implies that the LeuRS and tRNA^Leu^ can be horizontally acquired independently, and one does not seem to strongly restrict the evolution of the other. tRNAs are often involved in HGT, with many found in close proximity to mobile elements and genomic islands [43]. The lack of co-evolution we find for tRNA^Leu^ and LeuRS is in contrast to the finding that human but not *E. coli* TyrRS could complement yeast whose TyrRS gene had been disrupted [44]. However, this reported "species specificity" was found to be due to a small peptide element in TyrRS, whose modification allowed the switching of species-specific aminoacylation across taxonomic domains [44].

The horizontal acquisition of aaRS of the same specificity might reflect a stochastic event in the evolution of these ancient enzymes. Numerous HGT events have been reported in many aaRS of different amino acid specificity, and these involved transfers at different taxonomic levels [18-20]. If these enzymes have been undergoing horizontal transfers in many extant lineages without affecting the evolution of their cognate tRNA, we cannot exclude the possibility that these transfers occurred without any impact to their aminoacylation capacities. Hence, the frequent transfers and current distribution of aaRS may instead reflect neutral stochastic transfers [45] and replacements. On the other hand, different aaRS forms in some instances were shown to provide differential sensitivity to naturally occurring antibiotics (see discussion in [46]). The possibility of selection through antibiotic resistance is seen in duplicate forms of same-specificity aaRS in Bacteria [47-49], and was suggested as a possible driving force behind the replacement of aaRS homeoalleles [46]. However, this hypothesis still requires further investigation.

## Conclusions: the impact of ancient HGTs on present-day lineages

Shared ancestry alone cannot explain the incredible variety in the genetic material that lineages possess. With HGT, organismal evolution becomes a patchwork of genes from varied sources. As a lineage evolves through time, it receives and loses genes and gene fragments, not only from its close relatives through biased gene transfer [14,41] but also from the mobilome [50], from distantly related taxa, and from organisms that existed alongside a particular extant lineage and that have now become extinct. Ancient lineages are an important source of genetic diversity in extant taxa. Through vertical inheritance alone, this molecular diversity would likely take millions of years to achieve. When transfers occur from deep branching lineages and the recipient passes the horizontally acquired genetic material to its descendants, the ancient genes are maintained in the genomes or pan-genomes of existing organisms even if the original donors went extinct since the transfer occurred.

Highly divergent genes that have patchy distributions in extant lineages provide strong evidence for ancient HGTs, as observed not only in the haloarchaeal LeuRS but also reported in PylRS [12], SepRS [16], and the rare forms of Ser [14] and ThrRS [51]. In these cases, the genes still exhibit some degree of similarity with their homologs, allowing reliable phylogenetic reconstruction. More challenging are genes that have no recognizable homolog in other existing lineages as is the case for the thousands of intriguing orphan genes (or ORFans [52]) and gene families in extant genomes, whose evolutionary histories remain unaccounted for because they are present in only a small group of closely related organisms [53]. Assuming that most lineages that ever existed are now extinct [54,55], it is remarkable that evolution preserves some of these genes as molecular “fossils” [12]. However, the relative contributions from fast evolving phages and other components of the mobilome [56], unsampled or extinct lineages, and gene creation from previously non-coding DNA [57] remain to be ascertained.

The horizontal transmission of bacterial LeuRS to the Halobacteriales provides evidence for prokaryotic lineages that existed in the distant past and for their position in the Tree/Net of Life. The extremely long branch that in most molecular phylogenies separates LUCA from the base of the bacterial domain may have been populated by lineages that existed in the past. The results of the LeuRS analyses may be interpreted as evidence for the fourth domain of life, as reported in [58]. At present, we can only deduce few and tentative characteristics of these ancient, deep-branching lineages; however, future work may identify other donations made by these ancient lineages, possibly leading to a better characterization of these long extinct cousins of modern bacteria.

## Methods

Protein sequences of LeuRS from the three domains were retrieved by BLASTP searches of the non-redundant protein database and the BLAST microbial genome database from the National Center for Biotechnology Information (NCBI) website [59]. For the global phylogenetic analysis, 325 LeuRS sequences were used. Sequences were aligned using the MUSCLE algorithm [24] with default parameters. Maximum likelihood phylogenetic reconstruction of the LeuRS sequences was performed using PhyML v3.0 [60] with 100 bootstrap replicates, WAG [61] substitution model, estimated portions of invariable sites, four substitution-rate categories, estimated Γ distribution parameter, estimated amino acid frequencies, and NJ starting tree. Maximum likelihood distances were calculated using the programs PUZZLEBOOT v1.03 [62] and TREE-PUZZLE [63] using the WAG [61] + Γ + I model to calculate pairwise maximum likelihood distances and NEIGHBOR [64] for tree reconstruction. Posterior probability values were generated using MrBayes v. 3.1.2 [65], with a fixed WAG [61] amino acid substitution model using four rate categories approximating a Γ distribution, four chains and a random starting tree. We used a specified number of generations for each aaRS analyses (145,000 for the haloarchaeal LeuRS type A and 150,000 for the haloarchaeal LeuRS type B) sampling every 100th generation. The first 25% of the sampled generations were removed from the analysis as burn-in. Inspection of the convergence parameter and log likelihood values reveals that the exploration of the tree space has reached a plateau.

For comparison, we used a multilocus sequence analysis (MLSA) approach that comprises five housekeeping genes that encode for V-type ATP synthase subunit B (AtpB), elongation factor 2 (EF-2), DNA repair and recombination protein (RadA), RNA polymerase subunit B’ (RpoB’) and preprotein translocase subunit (SecY) [39]. The sequence data for each protein were partitioned using MrBayes v.3.1.2 [65] and phylogenetic estimates were calculated from the different data partitions. Tree reconstruction and calculation of posterior probability values were generated using MrBayes v.3.1.2 [65].

DNA sequences encoding the archaeal tRNA^Leu^ were obtained by BLASTN searches. tRNA tree reconstruction and bootstrapping were performed using PhyML v3.0 [60] with estimated portions of invariable sites, four substitution-rate categories, estimated ts/tv ratio, estimated Γ distribution parameter, estimated amino acid frequencies, BioNJ starting tree, 100 bootstrap replicates and GTR [66] nucleotide substitution model. Posterior probability values for the tRNA^Leu^ tree were generated using MrBayes v. 3.1.2 [65], with a fixed GTR nucleotide substitution model using four rate categories approximating a Γ distribution, four chains, a random starting tree, 50,000,000 generations sampling every 100th generation. The first 25% of the sampled generations were removed from the analysis as burn-in. Branch lengths and topologies of all phylograms were calculated with PhyML v3.0 [60]. Inspection of the convergence parameter and log likelihood values reveals that the exploration of the tree space has reached a plateau. The substitution models used for each approach were determined using ProtTest [67] and jModelTest [68].

For GARD analyses, a smaller dataset was selected, containing 13 bacterial, 9 haloarchaeal, and 14 sequences from other archaea (Additional file 2: Figure S2). Analyses were performed using GARD as implemented on the datamonkey [23]. The selection of the most appropriate substitution model (BLOSSUM62) was performed using the model selection program provided on the server. Using an alignment with MUSCLE as starting point, the sequences were realigned using SATé 2.03 [25] selecting MUSCLE for merger and ProGammaIBLOSSUM62 as substitution model. Protein structure files were downloaded from the RCSB Protein Data Bank [69] and visualized using the Swiss PDB viewer [70]. Positions in the *Thermus thermophilus* and *Pyrococcus horikoshii* structures corresponding to the identified breakpoint were identified using the multiple sequence alignment.

Genomic synteny among several members of the Halobacteriales and other Archaea was analyzed to identify the genes surrounding the *leuS* gene. This was done by aligning the genomes using the Integrated Microbial Genomes software tool provided by the U.S. Department of Energy Joint Genome Institute (http://img.jgi.doe.gov/cgi-bin/w/main.ci).

## Competing interests

The authors declare that they have no competing interests.

## Authors’ contributions

CPA carried out the phylogenetic analyses and drafted the manuscript. JPG performed the GARD analyses and generated the illustrations depicting the tRNA-LeuRS interactions. JPG and RTP participated in the design of this study and helped to draft the manuscript. All authors contributed to data analysis. All authors read and approved the final manuscript.

## Supplementary Material

Additional file 1 **Figure S1. Details of the LeuRS phylogenetic tree shown in Figure**1. Only bootstrap values above 50% and posterior probabilities above 0.50 are shown. Click here for file

Additional file 2 **Figure S2. Phylogenies calculated separately for the amino and carboxy terminal parts of the multiple sequence alignment.** Using a SATé alignment in GARD, we detected one significant breakpoint in the alignment. The two portions of the alignment were used separately for phylogenetic reconstruction. Panel A and C give phylogenies calculated from parts of the original SATé alignment, panel B and D give the phylogenies after the parts were realigned separately using MUSCLE, to avoid the possibility that a bias created in the original SATé alignment carries through to both portions of the multiple sequence alignment. Numbers give bootstrap support values calculated with PhyML, red branches indicate parts of the phylogeny leading to haloarchaeal sequences, branches with less than 80% bootstrap support are depicted as gray lines. Click here for file

Additional file 3 **Figure S3. Structure of archaeal (Panel A) and bacterial (Panel B) type LeuRSs complexed with tRNA**^**Leu**^**.** Panels A and B depict the structures of LeuRS from *Pyrococcus horikoshii* (1WZ2, [71]) and the *Thermus thermophilus* (2BYT [72]), respectively. The amino terminal portion of the protein that contains a strong phylogenetic signal is depicted in blue, the carboxy terminal part is less conserved between the domains is colored green. Atoms of side chains of amino acids within 6 Angstrom of the tRNA are depicted as space filling spheres, for the remainder of the protein only the alpha carbons of the protein backbone are depicted. Click here for file

Additional file 4 **Table S1. Percent identities of the haloarchaeal LeuRS.** The three-letter abbreviations are: *Haladaptatus* (Hap), *Halalkalicoccus* (Hac), *Haloarcula* (Har), *Halobacterium* (Hbt), *Haloferax* (Hfx), *Halogeometricum* (Hgm), *Halomicrobium* (Hmc), *Haloquadratum* (Hqr), *Halorhabdus* (Hrd), *Halorubrum* (Hrr), *Haloterrigena* (Htg), *Natrialba* (Nab). Hbt1 refers to *Halobacterium salinarum* and Hbt2 refers to *Halobacterium* sp. NRC-1. Comparisons between LeuRS A forms are in dark green, between B' forms in blue, and between B" forms in orange. Comparisons between B' and B" forms are in green, and between A and B forms in red. LeuRS.muscle.faa - Multiple sequence alignment in fasta format of the LeuRS sequences used for the phylogenetic reconstruction depicted in Figure 1. Click here for file
