# Supplementary figures and images for: Ancient origin of the divergent forms of leucyl-tRNA synthetases in the Halobacteriales
Source: BMC Evol Biol. 2012 Jun 13;12:85. doi: 10.1186/1471-2148-12-85 (PMC3436685; doi:10.1186/1471-2148-12-85)

0.5

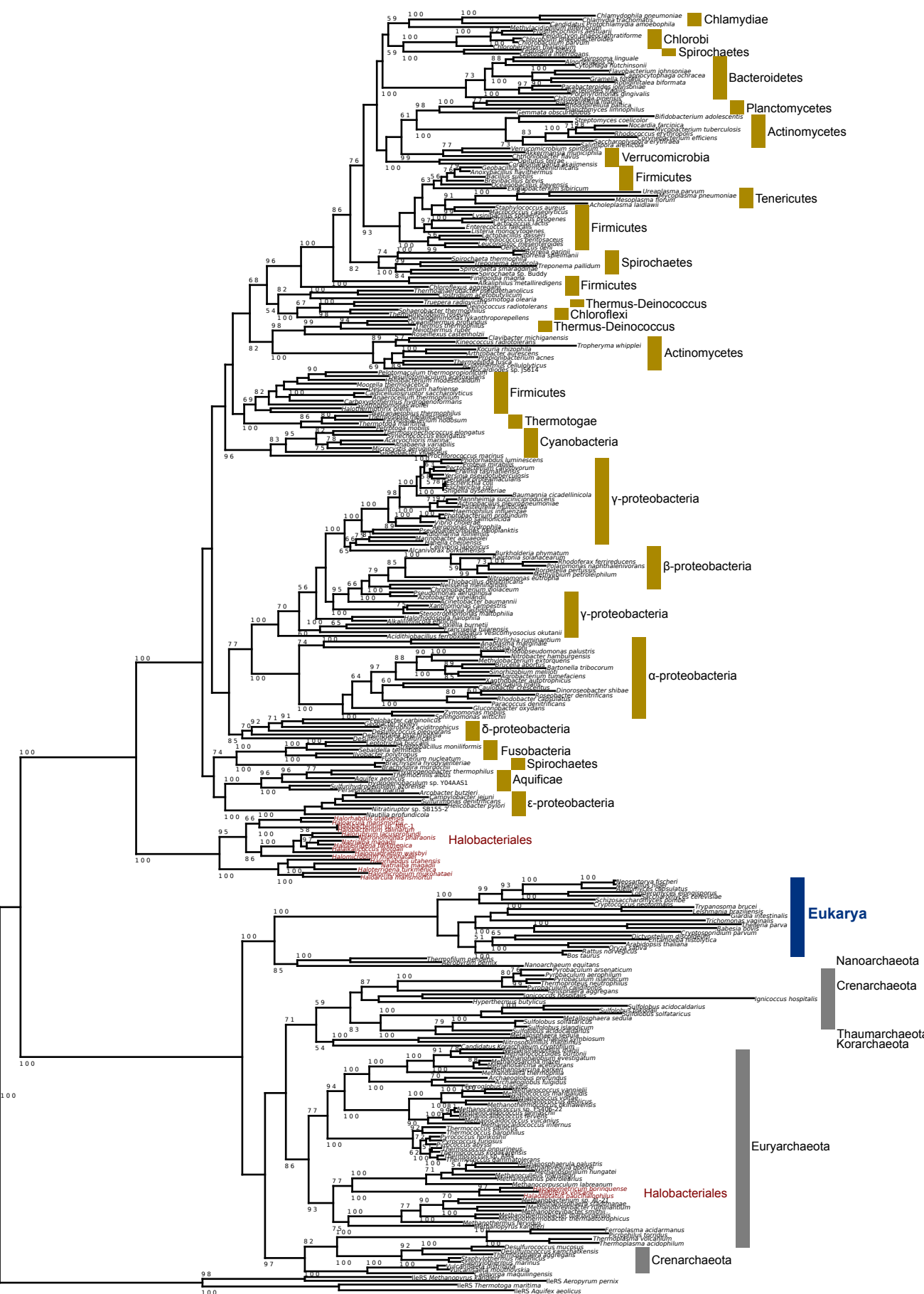

Supplement: Additional file 2 — Figure S2. Phylogenies calculated separately for the amino and carboxy terminal parts of the multiple sequence alignment. Using a SATé alignment in GARD, we detected one significant breakpoint in the alignment. The two portions of the alignment were used separately for phylogenetic reconstruction. Panel A and C give phylogenies calculated from parts of the original SATé alignment, panel B and D give the phylogenies after the parts were realigned separately using MUSCLE, to avoid the possibility that a bias created in the original SATé alignment carries through to both portions of the multiple sequence alignment. Numbers give bootstrap support values calculated with PhyML, red branches indicate parts of the phylogeny leading to haloarchaeal sequences, branches with less than 80% bootstrap support are depicted as gray lines. [file 1471-2148-12-85-S2.pdf]
