# Supplementary material for: Ancient origin of the divergent forms of leucyl-tRNA synthetases in the Halobacteriales
Source: BMC Evol Biol. 2012 Jun 13;12:85. doi: 10.1186/1471-2148-12-85 (PMC3436685; doi:10.1186/1471-2148-12-85)
Supplement: Additional file 3 — Figure S3. Structure of archaeal (Panel A) and bacterial (Panel B) type LeuRSs complexed with tRNALeu. Panels A and B depict the structures of LeuRS from Pyrococcus horikoshii (1WZ2, [71]) and the Thermus thermophilus (2BYT [72]), respectively. The amino terminal portion of the protein that contains a strong phylogenetic signal is depicted in blue, the carboxy terminal part is less conserved between the domains is colored green. Atoms of side chains of amino acids within 6 Angstrom of the tRNA are depicted as space filling spheres, for the remainder of the protein only the alpha carbons of the protein backbone are depicted. [file 1471-2148-12-85-S3.pdf]

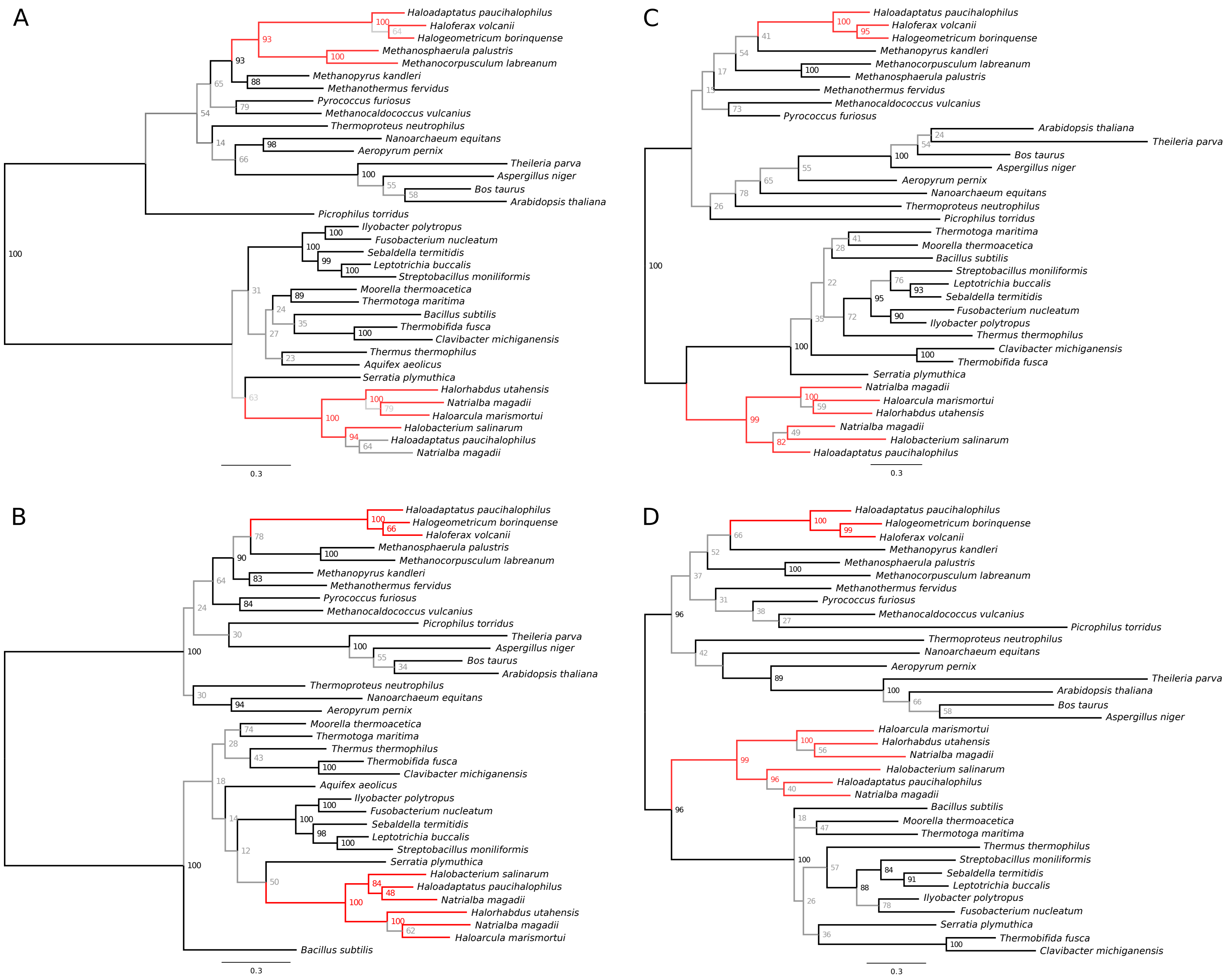

Figure S2. Phylogenies calculated separately for the amino and carboxy terminal parts of the multiple sequence alignment. Using a SATé alignment in GARD, we detected one significant breakpoint in the alignment. The two portions of the alignment were used separately for phylogenetic reconstruction. Panel A and C give phylogenies calculated from parts of the original SATé alignment, panel B and D give the phylogenies after the parts were realigned separately using MUSCLE, to avoid the possibility that a bias created in the original SATé alignment carries through to both portions of the multiple sequence alignment. Numbers give bootstrap support values calculated with PhyML, red branches indicate parts of the phylogeny leading to haloarchaeal sequences, branches with less than 80% bootstrap support are depicted as gray lines.
