# Supplementary material for: Ancient origin of the divergent forms of leucyl-tRNA synthetases in the Halobacteriales
Source: BMC Evol Biol. 2012 Jun 13;12:85. doi: 10.1186/1471-2148-12-85 (PMC3436685; doi:10.1186/1471-2148-12-85)
Supplement: Additional file 4 — Table S1. Percent identities of the haloarchaeal LeuRS. The three-letter abbreviations are: Haladaptatus (Hap), Halalkalicoccus (Hac), Haloarcula (Har), Halobacterium (Hbt), Haloferax (Hfx), Halogeometricum (Hgm), Halomicrobium (Hmc), Haloquadratum (Hqr), Halorhabdus (Hrd), Halorubrum (Hrr), Haloterrigena (Htg), Natrialba (Nab). Hbt1 refers to Halobacterium salinarum and Hbt2 refers to Halobacterium sp. NRC-1. Comparisons between LeuRS A forms are in dark green, between B' forms in blue, and between B" forms in orange. Comparisons between B' and B" forms are in green, and between A and B forms in red. LeuRS.muscle.faa - Multiple sequence alignment in fasta format of the LeuRS sequences used for the phylogenetic reconstruction depicted in Figure 1. [file 1471-2148-12-85-S4.pdf]

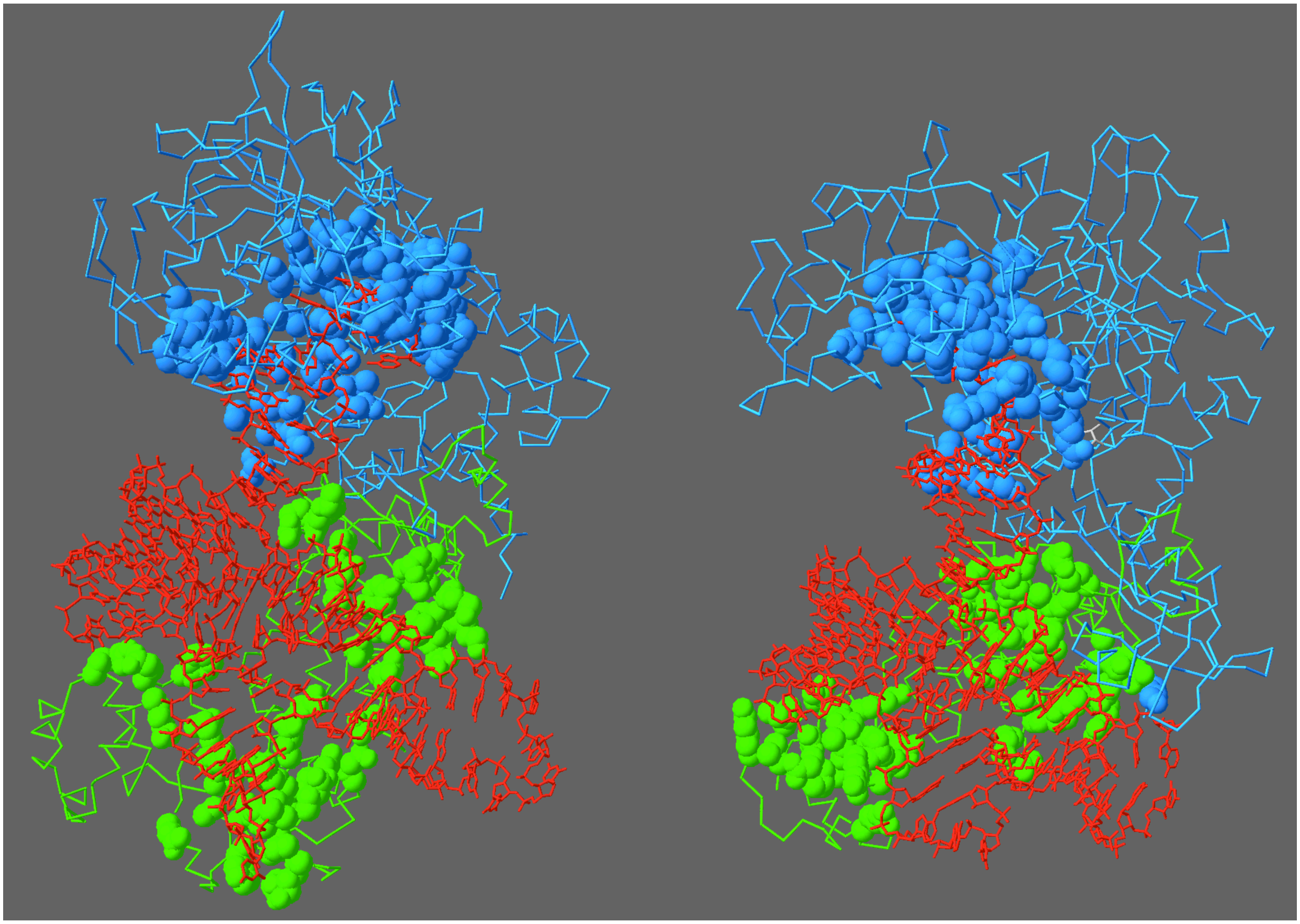

Figure S3. Structure of archaeal (Panel A) and bacterial (Panel B) type LeuRSs complexed with $tRNA^{Leu}$. Panels A and B depict the structures of LeuRS from *Pyrococcus horikoshii* (1WZ2) and the *Thermus thermophilus* (2BYT), respectively. The amino terminal portion of the protein that contains a strong phylogenetic signal is depicted in blue, the carboxy terminal part is less conserved between the domains and is colored green, and the tRNA is colored red. Atoms of side chains of amino acids within 6 Angstrom of the tRNA are depicted as space filling spheres, for the remainder of the protein only the alpha carbons of the protein backbone are depicted.
